# Development and Validation of the Chinese Physicians’ Reactions to Uncertainty Scale for Endoscopy: A Mixed‐Methods Approach

**DOI:** 10.1155/cjgh/2337892

**Published:** 2026-08-30

**Authors:** Yingnan Deng, Hanyue Ding, Wen Shi, Weiyang Zheng, Xiaowei Tang, Zhuo Yang, Hui Ding, Yunlu Feng, Aiming Yang, Dong Wu

**Affiliations:** ^1^ Department of Gastroenterology, Peking Union Medical College Hospital, Chinese Academy of Medical Sciences, Peking Union Medical College, Beijing, China, pumc.edu.cn; ^2^ School of Population Medicine and Public Health, Chinese Academy of Medical Sciences, Peking Union Medical College, Beijing, China, pumc.edu.cn; ^3^ Department of Gastroenterology, The Affiliated Hospital of Southwest Medical University, Luzhou, China, ahswmu.cn; ^4^ Department of Endoscope, General Hospital of Northern Theater Command, Shenyang, Liaoning, China, syjqzyy.com; ^5^ Department of Gastroenterology, People’s Hospital of Zhengzhou University (Henan Provincial People’s Hospital), Zhengzhou, Henan, China

**Keywords:** endoscopy, gastrointestinal, physicians, psychometrics, surveys and questionnaires, uncertainty

## Abstract

**Objectives:**

To develop and validate a Chinese Physicians’ Reactions to Uncertainty Scale for Endoscopy for assessing endoscopists’ reactions to uncertainty in clinical practice.

**Methods:**

Using a mixed‐methods design, we translated the original Physicians’ Reactions to Uncertainty scale and generated endoscopy‐specific items through focus‐group interviews. The draft scale was refined through Delphi consultation and then evaluated in an online survey of 360 digestive endoscopists from 204 hospitals in China. Item reduction was based on dispersion, critical ratio, and item–total correlation analyses. Construct validity was assessed using exploratory factor analysis and confirmatory factor analysis. Content validity was evaluated using item‐level and scale‐level content validity indices, and reliability was examined using Cronbach’s alpha and split‐half reliability.

**Results:**

The final scale comprised 16 items across four dimensions: concerns about uncertainty and bad outcomes, disclosing uncertainty to patients, disclosing mistakes to physicians, and endoscopy‐specific uncertainty responses and self‐efficacy. In exploratory factor analysis, four factors explained 73.474% of the total variance. Confirmatory factor analysis supported an acceptable four‐factor model fit (*χ* ^2^/df = 2.778, RMSEA = 0.098, IFI = 0.908, TLI = 0.931). Cronbach’s alpha was 0.818 for the overall scale and 0.801–0.932 for the subscales. Split‐half reliability was 0.919. Item‐level content validity indices ranged from 0.86 to 1.00, and the scale‐level content validity index was 0.96.

**Conclusions:**

The Chinese Physicians’ Reactions to Uncertainty Scale for Endoscopy is a reliable and valid instrument for assessing endoscopists’ reactions to uncertainty in China.

## 1. Introduction

In clinical practice, uncertainty refers to physicians’ awareness of incomplete, ambiguous, or unpredictable information during diagnosis, treatment, risk assessment, and communication with patients. Uncertainty is inherent in medicine and shapes both clinical decision‐making and patient outcomes. It can complicate quality assessment, increase anxiety for physicians and patients, and influence malpractice concerns and informed consent processes [1]. Uncertainty tolerance refers to cognitive, emotional, and behavioral responses to uncertainty and has been recognized as an important determinant of how physicians interpret and manage complex clinical situations [2]. Lower tolerance of uncertainty has been associated with greater healthcare resource utilization, lower well‐being, and different approaches to testing, referral, and adoption of new interventions [3–6]. These findings have strengthened interest in uncertainty as a meaningful target in medical education and professional development [7].

Several instruments have been used to assess physicians’ responses to uncertainty, including the Tolerance for Ambiguity scale, the Physicians’ Reactions to Uncertainty scale, and the Tolerance of Ambiguity in Medical Students and Doctors scale [8–10]. Among these instruments, the Physicians’ Reactions to Uncertainty scale has the strongest evidence base for validity in healthcare settings [11]. However, a validated Chinese version is not currently available. The reliability and applicability of uncertainty‐tolerance scales may vary across medical populations, specialties, and clinical contexts, supporting the need for specialty‐specific measures in highly technical fields [12, 13]. Most existing instruments were developed for general medical populations and emphasize broad cognitive or emotional reactions. They may therefore overlook how uncertainty is experienced in procedural specialties, where technical performance, immediate risk assessment, communication, and patient safety are closely connected.

Digestive endoscopy presents a distinctive form of clinical uncertainty. Endoscopists make real‐time decisions in technically demanding settings and often work under pressure related to procedural risk, patient discomfort, postoperative adverse events, and the introduction of emerging technologies [14–16]. Prior studies have suggested that endoscopist personality traits may be associated with procedural quality indicators and responses to quality‐improvement interventions [17–19]. Nevertheless, existing instruments do not fully capture the practical uncertainty experienced during endoscopy, such as incomplete visualization, unexpected findings, anatomical variation, patient discomfort, changing procedural conditions, and the need to decide immediately whether to continue, modify, or stop a procedure. These challenges link uncertainty directly to technical performance, communication, and patient safety, creating a clear need for an endoscopy‐specific assessment tool. A mixed‐methods design is particularly useful for developing such a tool because qualitative exploration can identify context‐specific psychological and behavioral responses, while expert consultation and quantitative psychometric testing can establish their clinical relevance and measurement quality [20, 21]. We therefore aimed to develop and validate a Chinese Physicians’ Reactions to Uncertainty Scale for Endoscopy derived from the original Physicians’ Reactions to Uncertainty scale 9. By combining established domains from the original scale with endoscopy‐specific items drawn from clinical experience, the study aimed to provide an assessment that better reflects the context of endoscopic practice, with a focus on concerns about uncertainty, communication behaviors, coping responses, and self‐efficacy in endoscopic practice.

## 2. Methods

### 2.1. Study Design

This study used a mixed‐methods design to develop and validate the Chinese Physicians’ Reactions to Uncertainty Scale for Endoscopy. The overall process was informed by methodological literature on questionnaire development, cross‐cultural adaptation, psychometric validation, and reporting standards for measurement instruments [20, 21].

### 2.2. Translation

After obtaining permission from the original author, we translated the original Physicians’ Reactions to Uncertainty scale into Chinese using a forward‐translation and back‐translation process based on the Brislin method [22]. Two bilingual native Chinese translators independently translated the original instrument from professional and linguistic perspectives. Discrepancies were discussed by the research team to produce a reconciled Chinese version. This version was then back‐translated by a doctoral‐level translator with overseas study experience and by a native English speaker, neither of whom had seen the original scale. The research team compared the back‐translated version with the original instrument, resolved semantic discrepancies, and refined wording to achieve conceptual and cultural equivalence.

### 2.3. Focus‐Group Interview

After a literature review [14–16, 23–25], the research team conducted focus‐group discussions with five experienced gastrointestinal endoscopists from Peking Union Medical College Hospital to generate endoscopy‐specific candidate items. All experts held associate or higher professional titles and had performed more than 10,000 endoscopic procedures. Ten endoscopy‐specific items were drafted to capture emotional responses and behavioral tendencies in uncertain endoscopic scenarios. Together with the 15 translated items from the original scale [9], these items formed a 25‐item draft version of the scale. Responses were scored on a 6‐point Likert scale.

### 2.4. Delphi Expert Consultation

A multidisciplinary expert panel was convened for Delphi consultation. In each round, panel members independently and anonymously rated item importance online using a 5‐point Likert scale. After each round, the median and quartiles for each item were fed back to the panel, and experts were invited to reconsider their ratings in the subsequent round. Four rounds of Delphi consultation were conducted at two‐week intervals. The fourth round was performed to confirm consensus, and its results were identical to those of the third round, indicating that no further item changes were required. Items were retained when the mean importance score was greater than 4.0, and the coefficient of variation was below 0.25.

### 2.5. Survey Participants and Sample Size

From April to May 2024, we used snowball sampling to recruit gastroenterologists to complete an online questionnaire [26]. The questionnaire included informed consent, demographic information, and the 22‐item post‐Delphi draft scale. Eligible participants were qualified endoscopists who could independently perform digestive endoscopy and endoscopic resection of colorectal polyps. Physicians who had retired, changed positions, or had not performed endoscopy during the previous year were excluded. To improve data quality, questionnaires with incomplete responses, duplicate submissions, extremely short completion times, or obvious patterned responses were excluded. The post‐Delphi draft scale contained 22 items. Based on the commonly used recommendation that factor analysis requires at least 5 to 10 participants per item [27], the minimum sample size was estimated as 110 participants using a 5:1 participant‐to‐item ratio. After allowing for approximately 10% invalid responses, the minimum required sample size was 122. In addition to the survey sample, seven independent experts in digestive endoscopy from Peking Union Medical College Hospital, who had not participated in the focus‐group interview or Delphi consultation, evaluated the content validity of the final scale using a 5‐point Likert scale.

### 2.6. Ethics

The study was approved by the Ethics Committee of the Chinese Academy of Medical Sciences & Peking Union Medical College (Identifier: K5268). According to the information sheet attached to the online questionnaire, participants provided informed consent by returning the completed survey. All procedures conformed to the Declaration of Helsinki.

### 2.7. Statistical Analysis

For the Delphi process, we used Kendall’s coefficient of concordance to evaluate agreement among experts and applied the predefined mean and coefficient‐of‐variation criteria for item retention. After collection of questionnaire data, items were screened using three methods: dispersion analysis, critical ratio analysis, and item–total correlation analysis. Items were retained when the standard deviation was greater than 0.9, the absolute critical ratio was at least 3.00 with *p* < 0.05, and the item–total correlation coefficient was at least 0.30. Construct validity was assessed using exploratory factor analysis and confirmatory factor analysis. Before exploratory factor analysis, we required a Kaiser–Meyer–Olkin value greater than 0.50 and a significant Bartlett’s test of sphericity (*p* < 0.05). Factors with eigenvalues greater than 1 were retained; each factor was required to contain at least two items, the cumulative variance explained had to exceed 50%, and item factor loadings had to exceed 0.50. Exploratory factor analysis was conducted using principal component analysis with varimax rotation. Confirmatory factor analysis was then performed using the remaining sample to test the factor structure. Convergent validity was evaluated using average variance extracted and composite reliability, with acceptable thresholds of greater than 0.50 and greater than 0.60, respectively. Content validity was assessed using the item‐level and scale‐level content validity indices. Reliability was examined using Cronbach’s alpha and split‐half reliability. Data were analyzed with SPSS Version 26.0, and structural equation modeling was performed using the lavaan package in R.

## 3. Results

### 3.1. Scale Development and Item Reduction

**FIGURE 1:**
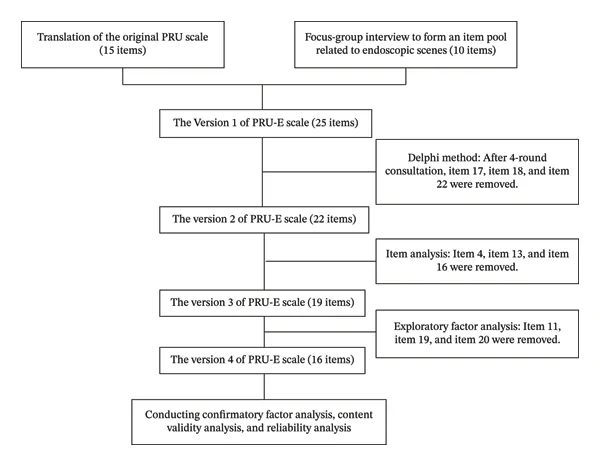
Development and validation process of the Chinese physicians’ reactions to uncertainty scale for endoscopy (PRU‐E).

The overall development and validation process is shown in Figure 1. Five experts participated in the focus‐group interview and generated 10 additional endoscopy‐specific items based on the original scale [9]. The characteristics of these experts are shown in Supporting Table 1. After translation of the original 15‐item scale and addition of 10 endoscopy‐specific items from the focus‐group interview, the draft scale contained 25 items (Supporting Table 2). These candidate items covered uncertainty related to high‐risk procedures, emergency patients, patient discomfort, postoperative complications, and emerging technologies. Following four rounds of Delphi consultation, three items (Items 17, 18, and 22) were removed, resulting in a 22‐item version. Item analysis then excluded three additional items: Item 16 did not meet the critical ratio criterion, and Items 4 and 13 showed item–total correlations below 0.30. The remaining 19 items were entered into exploratory factor analysis. Based on those results, three further items (Items 11, 19, and 20) were removed, and the final scale comprised 16 items across four dimensions.

### 3.2. Delphi Consultation

The Delphi panel included five gastroenterologists, one psychologist, and one public health scholar; four panelists were male. Six panelists were affiliated with hospitals, and one worked in a college. One expert held a senior title and four held associate titles (Supporting Table 3). Four Delphi rounds were completed. The fourth round produced the same results as the third round, confirming that consensus had been reached. Kendall’s coefficients of concordance ranged from 0.71 to 0.84, all with *p* < 0.001, indicating strong agreement among experts. After Delphi consultation, Items 17, 18, and 22 were removed from the draft scale (Supporting Table 4).

### 3.3. Characteristics of Survey Participants

After quality control, a total of 360 valid questionnaires from digestive endoscopists in 204 hospitals in China were included in the psychometric evaluation of the scale. Participants were randomly assigned to the EFA and CFA samples at an approximate 4:6 ratio. 150 participants were assigned to the exploratory factor analysis sample and 210 to the confirmatory factor analysis sample. The median age was 42 years; 49.2% were male, 86.7% specialized in digestive endoscopy, and 74.7% held a bachelor’s degree. Most participants worked in tertiary hospitals (85.0%) and had more than 10 years of clinical experience (53.6%). There were no significant differences between the exploratory and confirmatory samples in demographic or professional characteristics (all *p* > 0.05). Detailed participant characteristics are shown in Table 1.

**TABLE 1:** Characteristics of the participants (*n* = 360).

Variable	Total (*n* = 360)	EFA (*n* = 150)	CFA (*n* = 210)	Test statistic	*p* value
	*N* (%)/median (quartiles)	*N* (%)/median (quartiles)	*N* (%)/median (quartiles)		
Gender				*X* ^2^ = 1.739	0.201
Male	178 (49.0)	68 (45.3)	110 (52.4)		
Female	182 (51.0)	82 (54.7)	100 (47.6)		
Age	41 (38, 45)	41 (38, 45)	40.5 (38, 46)	*Z* = −0.873	0.383
Specialty				*X* ^2^ = 0.753	0.875
Endoscopy	312 (86.7)	131 (87.3)	181 (86.2)		
Others	48 (13.3)	19 (12.7)	29 (13.8)		
Years as a specialist	10 (6, 17)	10 (5, 17)	10 (6, 17)	*Z* = −0.495	0.620
Average endoscopic working time per week	20 (10, 30.5)	19 (10, 30)	20 (10, 35)	*Z* = −0.553	0.580
Annual endoscopy volume				*Z* = −0.618	0.537
< 300	72 (20.0)	34 (22.7)	38 (18.1)		
300∼< 600	166 (46.1)	58 (38.7)	108 (51.4)		
≥ 600	122 (33.9)	58 (38.7)	64 (30.5)		

Abbreviations: CFA, confirmatory factor analysis; EFA, exploratory factor analysis.

### 3.4. Item Analysis

In dispersion analysis, all items had standard deviations greater than 0.9. In critical ratio analysis, Item 16 failed to meet the selection criterion, whereas the absolute critical ratio values for the remaining items ranged from 3.355 to 13.771. In item–total correlation analysis, correlation coefficients ranged from 0.321 to 0.808 for all items except Items 4 and 13, which were therefore excluded (Supporting Table 5).

### 3.5. Construct Validity and Content Validity

Of the 360 questionnaires, 150 were used for exploratory factor analysis and 210 for confirmatory factor analysis. Before exploratory factor analysis, the Kaiser–Meyer–Olkin value was 0.826, and Bartlett’s test of sphericity was significant (*χ* ^2^ = 1792.41, df = 120, *p* < 0.001), indicating that the data were suitable for factor analysis. Exploratory factor analysis was conducted using principal component analysis with varimax rotation and a factor‐loading threshold greater than 0.50. Items 11, 19, and 20 were removed. Four common factors with eigenvalues greater than 1 were retained (Supporting Figure 1), explaining 73.474% of the total variance (Supporting Table 6). The final 16‐item scale comprised four dimensions: concerns about uncertainty and bad outcomes (Items 1, 2, 3, 5, 6, 7, and 8), disclosing uncertainty to patients (Items 9, 10, and 12), disclosing mistakes to physicians (Items 14 and 15), and endoscopy‐specific uncertainty responses and self‐efficacy (Items 21, 23, 24, and 25). Factor 1 reflected emotional concern, anxiety, and fear of adverse consequences under uncertainty. Factor 2 reflected physicians’ willingness to disclose uncertainty to patients. Factor 3 reflected reluctance to disclose missed diagnoses or mistakes to colleagues. Factor 4 reflected endoscopy‐specific uncertainty responses and self‐efficacy across high‐risk procedures, patient discomfort, emerging technologies, and complication management. Factor loadings are shown in Table 2. Items 9, 10, 12, 24, and 25 were reverse‐scored.

**TABLE 2:** The final PRU‐E scale and factor loadings of each item in four common factors.

No.	Items	F1	F2	F3	F4
*F1. Concerns about uncertainty and bad outcomes*					
1	I usually feel anxious when I am not sure of a diagnosis.	0.710			
2	I find the uncertainty involved in patient care disconcerting.	0.835			
3	Uncertainty in patient care makes me uneasy.	0.879			
5	The uncertainty of patient care often troubles me.	0.852			
6	When I am uncertain of a diagnosis, I imagine all sorts of bad scenarios—patient dies, patient sues, etc.	0.819			
7	I fear being held accountable for the limits of my knowledge.	0.873			
8	I am concerned about the potential for malpractice when I am unaware of a patient’s diagnosis.	0.841			

*F2. Disclosing uncertainty to patients*					
9	When physicians are uncertain of a diagnosis, they should share this information with their patients.^a^		0.780		
10	I always share my uncertainty with my patients.^a^		0.632		
12	Sharing my uncertainty improves my relationship with my patients.^a^		0.673		

*F3. Disclosing mistakes to physicians*					
14	I almost never tell other physicians about diagnoses I have missed.			0.799	
15	I never tell other physicians about patient care mistakes I have made.			0.748	

*F4. Endoscopy-specific uncertainty responses and self-efficacy*					
21	When performing endoscopies with a high risk of bleeding in outpatient departments, I imagine bad scenarios involving serious complications such as bleeding, perforation, and shock.				0.560
23	When patients experience pain or discomfort during endoscopies, I will stop invasive procedures.				0.548
24	I prefer to use emerging technologies in endoscopies.^a^				0.682
25	When patients encounter complications after endoscopies, I could manage them calmly.^a^				0.593

^a^ltems that are reverse‐scored.

### 3.6. Confirmatory Factor Analysis

**FIGURE 2:**
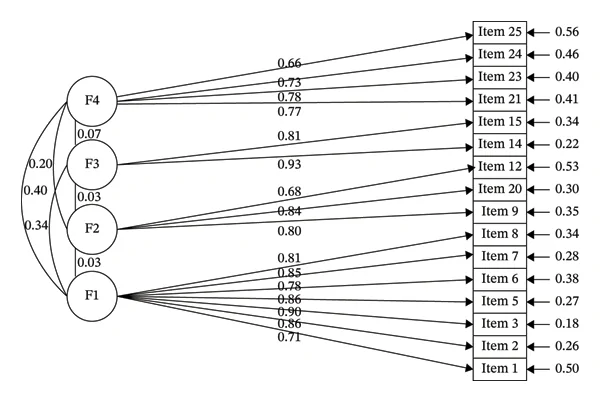
Confirmatory factor analysis model of the four‐factor PRU‐E scale.

Confirmatory factor analysis yielded the following model‐fit indices: *χ* ^2^/df = 2.778, RMSEA = 0.098, GFI = 0.830, NFI = 0.864, IFI = 0.908, and TLI = 0.931. These values indicated an acceptable, although not optimal, fit for the four‐factor model (Figure 2). Standardized factor loadings ranged from 0.64 to 0.93 and were statistically significant. Standardized regression coefficients ranged from 0.765 to 0.901. Modification indices ranged from < 0.001 to 24.78, and six values exceeded 10. The largest modification index suggested a residual covariance between Items 2 and 3 (MI = 24.78). Among the six indices above 10, five involved residual covariances, mainly between semantically similar items within Factor 1, while one suggested that reverse‐scored Item 9 might also load on Factor 4 (MI = 11.68). These findings were considered more likely to reflect similarities in item wording or response format than a fundamental problem with the four‐factor structure. Because these possible changes were identified only after the model had been fitted, adding them could have made the model overly dependent on the current sample. We therefore retained the original model to preserve its simplicity, theoretical clarity, and consistency with the domains of the original Physicians’ Reactions to Uncertainty scale. The average variance extracted values were greater than 0.50, and the composite reliability values were greater than 0.60, indicating acceptable convergent validity.

### 3.7. Content Validity

The characteristics of the seven content‐validity experts are shown in Supporting Table 7. The item‐level content validity index ranged from 0.86 to 1.00, and the scale‐level content validity index was 0.96, indicating strong content validity.

### 3.8. Reliability

Cronbach’s alpha coefficients for the four dimensions ranged from 0.801 to 0.932, and the Cronbach’s alpha for the overall scale was 0.818 (Table 3). The split‐half reliability coefficient for the full scale was 0.919, and all four dimensions exceeded 0.70. These findings indicate satisfactory internal consistency and reliability.

**TABLE 3:** Internal consistency reliability and split‐half reliability of the PRU‐E scale.

Dimension (number of entries)	Cronbach’s ɑ coefficient	Split‐half reliability
Concerns about uncertainty and bad outcomes (7)	0.932	0.949
Disclose uncertainty to patients (3)	0.823	0.757
Disclose mistakes to physicians (2)	0.900	0.900
Endoscopy‐specific uncertainty responses and self‐efficacy (4)	0.801	0.821
Summary	0.818	0.919

## 4. Discussion

We developed and validated a Chinese Physicians’ Reactions to Uncertainty Scale for Endoscopy using a mixed‐methods approach that combined translation, focus‐group interviews, Delphi consultation, and psychometric testing in a national sample of digestive endoscopists. The final instrument contained 16 items across four dimensions and demonstrated acceptable construct validity, strong content validity, and good reliability.

The current study supports the adaptation of the original Physicians’ Reactions to Uncertainty scale [9] to the setting of digestive endoscopy. The retained structure preserved the core domains of concern about uncertainty and adverse outcomes, reluctance to disclose uncertainty to patients, and reluctance to disclose mistakes to physicians, while adding a domain focused on endoscopy‐specific uncertainty responses and self‐efficacy. This extension is clinically important because endoscopy involves immediate technical decisions, concern about procedural harm, patient discomfort, and rapid judgment in the context of evolving technologies [14–16].

Several items from the original scale were removed during adaptation and validation. This likely reflects both the specific workflow of endoscopic practice and the cultural context in which Chinese physicians communicate uncertainty. In highly procedural settings, physicians may frame uncertainty less as an abstract cognitive state and more as a practical, action‐oriented challenge linked to technical performance and patient safety [28, 29]. Previous work has also suggested partial overlap between anxiety due to uncertainty and concern about adverse outcomes, which may explain why these constructs clustered together in the present analysis [30].

The CFA supported the four‐factor structure, although some fit indices were not optimal. In particular, the RMSEA was relatively high, and the GFI was below the conventional threshold. Several factors may explain this finding. First, Factor 1 contained seven items covering diagnostic anxiety, concerns about malpractice, accountability, and catastrophizing, which may have introduced conceptual heterogeneity. Second, the CFA sample size was moderate for a four‐factor model. Third, the final instrument combined translated items from the original PRU scale with newly developed endoscopy‐specific items, which may have increased model complexity. Several modification indices suggested local associations between closely related items, especially within Factor 1. These associations may have resulted from overlapping wording or from the use of reverse‐scored items. We did not revise the model on the basis of these post hoc findings because doing so might improve fit only in the present sample and reduce the model’s generalizability. Retaining the original structure also helped preserve its theoretical meaning and comparability with the original PRU scale.

The fourth dimension, endoscopy‐specific uncertainty responses and self‐efficacy, includes emotional concern in high‐risk procedures, behavioral responses to patient discomfort, openness to emerging technologies, and perceived competence in managing complications. After psychometric screening, the retained items emphasized the forms of uncertainty most closely tied to real‐time endoscopic practice. These findings are consistent with prior studies showing that physician traits and communication styles can influence procedural quality, intervention uptake, and patient‐centered care [17–19, 31, 32].

The scale may have several practical applications. It could be used to identify endoscopists who experience greater uncertainty‐related stress, to evaluate educational or supportive interventions, and to explore associations between uncertainty responses and procedural outcomes. Physician burnout is prevalent and has implications for professional well‐being, while both physician‐directed and organization‐directed interventions can produce modest improvements [33, 34]. In procedurally demanding settings, objective diagnostic aids and navigation technologies can reduce diagnostic or technical uncertainty and improve decision support or procedural outcomes, illustrating the broader patient‐safety relevance of uncertainty management across specialties [35, 36]. Such work may ultimately help clarify how uncertainty tolerance relates to physician well‐being, clinical performance, and patient safety in digestive endoscopy [37].

This study has strengths and limitations. Strengths include the mixed‐methods design, the integration of expert review and empirical validation, and the development of a specialty‐specific instrument rather than relying on a general uncertainty scale. Although the Delphi panel was relatively small, it included gastroenterology, psychology, and public health experts with relevant clinical or methodological experience, and the high Kendall’s coefficients suggested strong agreement among panel members. Nevertheless, future studies with larger and more geographically diverse expert panels may further refine the scale. One limitation is that participants were recruited online through snowball sampling. Because this approach relies on professional networks, some groups of endoscopists may have been overrepresented, which could have introduced selection bias [26]. The absence of an established external criterion also limits score interpretation. Most participants were from tertiary hospitals, which may limit generalizability to secondary hospitals, community settings, private practice, rural regions, and less‐experienced endoscopists. Endoscopists in different practice settings may face different workloads, resource availability, patient populations, and uncertainty profiles. Test–retest reliability was not assessed in the present study; therefore, the temporal stability of the PRU‐E scale remains to be evaluated. Additional studies should evaluate temporal stability, external validity, and performance in other regions and practice settings.

A preliminary version of this work was presented as a poster abstract at Asian Pacific Digestive Week 2026 and published in Journal of Gastroenterology and Hepatology [38]. The present full‐length manuscript substantially extends that conference abstract by providing the complete mixed‐methods development process, the full item‐reduction workflow, comprehensive psychometric analyses based on the national survey sample, and a detailed interpretation of the findings. Overall, the Chinese Physicians’ Reactions to Uncertainty Scale for Endoscopy appears to be a useful instrument for assessing endoscopists’ reactions to uncertainty in China.

## Author Contributions

Yingnan Deng and Hanyue Ding contributed equally to this work. Yunlu Feng, Aiming Yang, and Dong Wu conceived and supervised the study. Yingnan Deng, Hanyue Ding, Wen Shi, Weiyang Zheng, Xiaowei Tang, Zhuo Yang, and Hui Ding contributed to investigation, expert coordination, and data collection. Yingnan Deng and Hanyue Ding performed the formal analysis and drafted the manuscript. Yunlu Feng, Aiming Yang, and Dong Wu critically revised the manuscript for important intellectual content.

## Funding

The authors declare that financial support was received for the research, authorship, and/or publication of this article. This study was supported by the Science and Technology Planning Project of Tibet Autonomous Region (XZ202501JD0021) and Clinical Research Excellence Program for Research‐Oriented Wards (BRWEP2024W034010103).

## Disclosure

All authors interpreted the data, approved the final version of the manuscript, and agree to be accountable for all aspects of the work.

## Ethics Statement

This study was approved by the Ethics Committee of the Chinese Academy of Medical Sciences & Peking Union Medical College (Identifier: K5268). Informed consent was obtained electronically from all participants before questionnaire completion.

## Conflicts of Interest

The authors declare no conflicts of interest.

## Supporting Information

Additional supporting information can be found online in the Supporting Information section.

## Supporting information


**Supporting Information** The supporting information includes one figure and seven tables. Supporting Figure 1 provides the scree plot from the exploratory factor analysis. Supporting Table 1 summarizes the characteristics of the experts who participated in the focus‐group interview, and Supporting Table 2 lists the 25‐item Version 1 of the PRU‐E scale after translation and item generation. Supporting Table 3 describes the Delphi consultation experts, while Supporting Table 4 details the results of the Delphi consultation and item screening process. Supporting Table 5 reports the item analysis results, including dispersion analysis, critical ratio testing, and item–total correlations. Supporting Table 6 summarizes the common factors extracted from exploratory factor analysis, and Supporting Table 7 provides the characteristics of the experts involved in the content validity analysis.

## Data Availability

The data that support the findings of this study are available from the corresponding author upon request.
