# Supplementary material for: Development and Validation of the Chinese Physicians’ Reactions to Uncertainty Scale for Endoscopy: A Mixed‐Methods Approach
Source: Can J Gastroenterol Hepatol. 2026 Aug 30;2026:2337892. doi: 10.1155/cjgh/2337892 (PMC13527232; doi:10.1155/cjgh/2337892)
Supplement: Supplementary file 1 [file CJGH-2026-2337892-s001.doc]

Supplementary Figure 1. Scree plot for the exploratory factor analysis

Supplementary Table 1. Characteristics of experts for the focus-group interview (n = 5)

| No. | Age | Gender | Degree | Specialty | Professional title | Workplace | Working life |
| --- | --- | --- | --- | --- | --- | --- | --- |
| 1 | 49 | male | MD | Gastroenterologist | Senior | General hospital | 22 |
| 2 | 50 | male | MD | Gastroenterologist | Senior | General hospital | 21 |
| 3 | 45 | female | MD | Gastroenterologist | Associate | General hospital | 19 |
| 4 | 38 | male | MD | Gastroenterologist | Associate | General hospital | 16 |
| 5 | 44 | male | MD | Gastroenterologist | Associate | General hospital | 15 |

Supplementary Table 2. The version 1 of the PRU-E scale

| **No.** | **Item content** |
| --- | --- |
|  |  |
| 1 | I usually feel anxious when I am not sure of a diagnosis. |
| 2 | I find the uncertainty involved in patient care disconcerting. |
| 3 | Uncertainty in patient care makes me uneasy. |
| 4 | I am quite comfortable with the uncertainty in patient care. |
| 5 | The uncertainty of patient care often troubles me. |
| 6 | When I am uncertain of a diagnosis, I imagine all sorts of bad scenarios- patient dies, patient sues, etc .... |
| 7 | I fear being held accountable for the limits of my knowledge. |
| 8 | I am concerned about the potential for malpractice when I am unaware of a patient's diagnosis. |
| 9 | When physicians are uncertain of a diagnosis, they should share this information with their patients. |
| 10 | I always share my uncertainty with my patients. |
| 11 | If I shared all of my uncertainties with my patients, they would lose confidence in me. |
| 12 | Sharing my uncertainty improves my relationship with my patients. |
| 13 | I prefer patients not know when I am uncertain of what treatments to use. |
| 14 | I almost never tell other physicians about diagnoses I have missed. |
| 15 | I never tell other physicians about patient care mistakes I have made. |
| 16 | I feel uneasy when performing endoscopic examinations with a high risk of bleeding or perforation in outpatient departments. |
| 17 | When conducting endoscopic examinations for critical patients in emergency departments, I usually feel at ease. |
| 18 | I feel nervous when patients express discomfort during endoscopic procedures. |
| 19 | I feel anxious when using unfamiliar technologies during endoscopies. |
| 20 | When a patient experiences complications after endoscopy treatment, I often feel anxious and have ongoing concerns. |
| 21 | When performing endoscopies with high risk in outpatient departments, I imagine bad scenarios involving serious complications such as bleeding, perforation and shock. |
| 22 | I avoid performing endoscopies on critical patients in the emergency department. |
| 23 | When patients experience pain or discomfort during endoscopies, I will stop invasive procedures. |
| 24 | I prefer to use emerging technologies in endoscopies. |
| 25 | When patients encounter complications after endoscopies, I can manage them calmly. |

| No. | Age | Gender | Degree | Specialty | Professional title | Workplace | Working life |
| --- | --- | --- | --- | --- | --- | --- | --- |
| 1 | 48 | male | MD | Gastroenterologist | Senior | General hospital | 22 |
| 2 | 45 | female | MD | Gastroenterologist | Associate | General hospital | 19 |
| 3 | 42 | male | MD | Gastroenterologist | Associate | General hospital | 16 |
| 4 | 38 | male | MD | Gastroenterologist | Intermediate | General hospital | 12 |
| 5 | 42 | male | MD | Gastroenterologist | Intermediate | General hospital | 16 |
| 6 | 44 | female | MD | Psychologist | Associate | General hospital | 18 |
| 7 | 32 | female | PhD | Public health | Intermediate | Comprehensive university | 5 |

Supplementary Table 3. Characteristics of experts for the Delphi expert consultation (n = 7)

Supplementary Table 4. The results of the Delphi consultation

| No. | Round 1 | | Round 2 | | Round 3^c^ | |
| --- | --- | --- | --- | --- | --- | --- |
|  | mean^a^ | CV | mean^a^ | CV | mean^a^ | CV |
| 1 | 4.143 | 0.217 | 4.571 | 0.117 | 4.571 | 0.117 |
| 2 | 4.286 | 0.176 | 4.286 | 0.176 | 4.286 | 0.176 |
| 3 | 4.286 | 0.176 | 4.429 | 0.121 | 4.429 | 0.121 |
| 4 ^b^ | 4.143 | 0.217 | 4.143 | 0.217 | 4.143 | 0.217 |
| 5 | 4.429 | 0.178 | 4.429 | 0.178 | 4.429 | 0.178 |
| 6 | 4.429 | 0.178 | 4.429 | 0.178 | 4.429 | 0.178 |
| 7 | 4.429 | 0.178 | 4.429 | 0.178 | 4.429 | 0.178 |
| 8 | 4.000 | 0.204 | 4.000 | 0.204 | 4.000 | 0.204 |
| 9 ^b^ | 4.143 | 0.217 | 4.286 | 0.176 | 4.286 | 0.176 |
| 10 ^b^ | 4.143 | 0.167 | 4.143 | 0.167 | 4.143 | 0.167 |
| 11 | 4.143 | 0.167 | 4.143 | 0.167 | 4.143 | 0.167 |
| 12 ^b^ | 4.429 | 0.178 | 4.429 | 0.178 | 4.429 | 0.178 |
| 13 | 4.429 | 0.178 | 4.429 | 0.178 | 4.429 | 0.178 |
| 14 | 4.286 | 0.176 | 4.286 | 0.176 | 4.286 | 0.176 |
| 15 | 4.143 | 0.217 | 4.143 | 0.217 | 4.143 | 0.217 |
| 16 | 4.429 | 0.178 | 4.429 | 0.178 | 4.429 | 0.121 |
| **17 ^b^** | **3.714** | **0.403** | **3.714** | **0.300** | **3.714** | **0.131** |
| **18** | **4.143** | **0.217** | **4.000** | **0.204** | **3.714** | **0.131** |
| 19 | 4.143 | 0.217 | 4.143 | 0.217 | 4.286 | 0.114 |
| 20 | 4.143 | 0.217 | 4.143 | 0.217 | 4.143 | 0.217 |
| 21 | 3.857 | 0.380 | 4.286 | 0.176 | 4.143 | 0.217 |
| **22** | **3.857** | **0.315** | **3.571** | **0.273** | **3.429** | **0.156** |
| 23 | 3.857 | 0.315 | 4.143 | 0.217 | 4.571 | 0.117 |
| 24 ^b^ | 4.286 | 0.176 | 4.286 | 0.176 | 4.286 | 0.114 |
| 25 ^b^ | 3.714 | 0.403 | 4.286 | 0.176 | 4.571 | 0.117 |

CV coefficient of variation. ^a^: Mean importance score. ^b^: Items that are reverse scored. ^c^: The results of the fourth and third rounds were the same. The underlined mean importance score is less than 4 and the corresponding items are screened out.

Supplementary Table 5. The results of the item analyses

| No. | Dispersion degree analysis | Critical ratio method | | Correlation coefficient of the item and the total score |
| --- | --- | --- | --- | --- |
|  | Standard deviation | t value | p |  |
| 1 | 1.534 | -11.016 | ＜0.001 | 0.643 |
| 2 | 1.482 | -13.771 | ＜0.001 | 0.766 |
| 3 | 1.446 | -12.843 | ＜0.001 | 0.808 |
| **4** | **1.143** | **-12.405** | **＜0.001** | **0.157** |
| 5 | 1.566 | -4.869 | ＜0.001 | 0.773 |
| 6 | 1.510 | -5.589 | ＜0.001 | 0.784 |
| 7 | 1.483 | -11.988 | ＜0.001 | 0.845 |
| 8 | 1.377 | -15.916 | ＜0.001 | 0.799 |
| 9 | 1.194 | -14.825 | ＜0.001 | 0.321 |
| 10 | 1.495 | -4.436 | ＜0.001 | 0.388 |
| 11 | 1.498 | -4.666 | ＜0.001 | 0.430 |
| 12 | 1.332 | -3.355 | ＜0.001 | 0.406 |
| **13** | **1.432** | **-5.673** | **＜0.001** | **0.298** |
| 14 | 1.147 | -6.732 | ＜0.001 | 0.658 |
| 15 | 1.179 | -7.471 | ＜0.001 | 0.615 |
| **16** | **1.555** | **-0.988** | **0.326** | **0.338** |
| 19 | 1.447 | -6.245 | ＜0.001 | 0.683 |
| 20 | 1.341 | -9.365 | ＜0.001 | 0.565 |
| 21 | 1.373 | -7.258 | ＜0.001 | 0.738 |
| 23 | 1.162 | -10.514 | ＜0.001 | 0.636 |
| 24 | 1.040 | -6.712 | ＜0.001 | 0.712 |
| 25 | 1.092 | -8.395 | ＜0.001 | 0.583 |

The underlined items could not meet the filtering criteria and are removed.

Supplementary Table 6. The common factors extracted from the exploratory factor analysis

| Factor | Initial eigenvalue | | | Sum of the rotating load quares | | |
| --- | --- | --- | --- | --- | --- | --- |
|  | Total | Variance interpretation rate (%) | Cumulative contribution rate (%) | Total | Variance interpretation rate (%) | Cumulative contribution rate (%) |
| 1 | 5.677 | 35.481 | 35.481 | 5.038 | 31.486 | 31.486 |
| 2 | 2.682 | 16.764 | 52.245 | 2.575 | 16.096 | 47.582 |
| 3 | 1.951 | 12.196 | 64.44 | 2.254 | 14.088 | 61.671 |
| 4 | 1.445 | 9.034 | 73.474 | 1.889 | 11.804 | 73.474 |

Supplementary Table 7. Characteristics of experts for the content validity analysis (n = 7)

| No. | Age | Gender | Degree | Specialty | Professional title | Workplace | Working life |
| --- | --- | --- | --- | --- | --- | --- | --- |
| 1 | 56 | male | MD | Gastroenterologist | Senior | General hospital | 29 |
| 2 | 50 | male | MD | Gastroenterologist | Senior | General hospital | 24 |
| 3 | 48 | female | MD | Gastroenterologist | Associate | General hospital | 22 |
| 4 | 39 | male | MD | Gastroenterologist | Associate | General hospital | 13 |
| 5 | 34 | female | MD | Gastroenterologist | Intermediate | General hospital | 8 |
| 6 | 36 | male | MD | Gastroenterologist | Intermediate | General hospital | 10 |
| 7 | 41 | male | MD | Gastroenterologist | Intermediate | General hospital | 15 |
